# Variation in the Content of Bioactive Compounds in Infusions Prepared from Different Parts of Wild Polish Stinging Nettle (*Urtica dioica* L.)

**DOI:** 10.3390/molecules27134242

**Published:** 2022-06-30

**Authors:** Magdalena Jeszka-Skowron, Agnieszka Zgoła-Grześkowiak, Robert Frankowski, Tomasz Grześkowiak, Anna Maria Jeszka

**Affiliations:** 1 Institute of Chemistry and Technical Electrochemistry, Poznan University of Technology, Berdychowo 4, 60-965 Poznań, Poland; agnieszka.zgola-grzeskowiak@put.poznan.pl (A.Z.-G.); robert.frankowski@put.poznan.pl (R.F.); civ@tlen.pl (T.G.); 2 Institute of Socio-Economics, Poznan University of Economics and Business, Al. Niepodległości 10, 61-875 Poznań, Poland; anna.jeszka@ue.poznan.pl

**Keywords:** nettle, antioxidant activity, phenolic compounds, trigonelline, short-chain organic acids, LC–MS/MS, capillary isotachophoresis

## Abstract

Nettle is a common plant that offers many health benefits and is grown all over the world. The content of active compounds in roots, stems, and leaves was determined based on the extraction procedure optimized using the Central Composite Design. Flavonols, phenolic acids, trigonelline, nicotinamide, nicotinic acids, and short-chain organic acids were determined with the use of LC–MS/MS and capillary isotachophoresis. Trigonelline, which was not previously reported in the roots and stems of nettle, was found in all parts of the plant and considerable variations in its content were observed (2.8–108 µg g^−1^). Furthermore, the Principal Component Analysis taking into account more variables demonstrated differences in the content of bioactive components between roots and aerial parts of nettle.

## 1. Introduction

The knowledge of the beneficial properties of nettle (*Urtica dioica* L.) dates back to the time of Hippocrates. Nettle and its subspecies grow in temperate and tropical climates in Europe, Asia, North and South America, and Africa. Although, it is a common wild-growing species that is mostly considered a weed in agriculture. It grows widely in natural sites (roadside ditches, forests, and meadows), but due to its commercial potential, it is increasingly being cultivated in plantations as a part of industrial production (in the case of agricultural cultivation, swathing takes place 2–5 times a year). However, cultivation still requires the definition of a production model to maximize the use of plant biomass and improve efficiency in this regard, which is a prerequisite to increase income and attract investors/farmers [1,2]. In addition, improving soil quality would allow for better yields. Therefore, scientists have also conducted studies on the effect of nitrogen fertilization and the effect of culture media on the rooting of nettle [3,4].

To maintain a high quality of the raw material, the plants are dried in drying houses, packed, and stored in adapted storage facilities. This method of stock-keeping maintains the high content of bioactive compounds and protects against blackening or mold caused by bacteria and fungi [5]. Due to its rich chemical composition [6] as a plant raw material with a wide range of uses, nettle has attracted the attention of practitioners and academics, considering the composition and bioactive properties contained in different parts of the plant, leaf, stem, and root that are used in various fields. In livestock practice, leaves are used as an additive for animal feed, especially for young animals, in gardens and orchards as an insecticide and for fertilization, and in the cosmetic industry for the treatment of scalp conditions and hair loss. There has been a return to the idea of using nettle in the textile industry, where it was used until World War II [7].

However, above all, nettle is used in human nutrition and dietary supplements due to its rich chemical composition and the growing interest of consumers in healthy and bioactive foods [8]. The healthy lifestyle and eating habits presented by social media have popularized nettle as a food additive, especially in infusions, smoothies, soups, salads, rice or pasta dishes, etc. This has resulted in new trends in consumer behavior and an increased demand for this plant. Nettle leaves contain chlorophylls, carotenoids, vitamins, and phenolic compounds, mainly rutin, 5-caffeoylquinic (5-CQA), and 2-O-caffeoylmalic acid [9,10]. The other phenolic derivatives of quinic acid include 3-caffeoylquinic, 4-caffeoylquinic acids, and O-feruloylquinic acid. Apart from phenolic acids (hydroxycinnamic and hydroxybenzoic acids), nettle leaves can be a source of almost all the groups of flavonoids (e.g., flavones, flavonols, isoflavones, anthocyanins, flavan-3ols, flavanones, coumarins, and lignans) depending on the extraction process and solvent selection [6,11,12,13]. Due to the content of bioactive compounds with medicinal effects, it is widely used in phytotherapy (herbal medicine) [5,14,15].

Contemporary research in this area has documented antihyperglycemic [16], diuretic [17], antifungal [18], and antibacterial [19] properties. Nettle extracts have been shown to treat arthritis [20] and breast cancer [21]. Currently, nettle is most often used as a base for the production of drugs and dietary supplements, as well as cosmetics. Nevertheless, finding new applications and improving the quality of products based on herbal plants, including nettle, requires further analysis in terms of their active components.

The purpose of this study was to analyze the aerial- and underground parts of Polish wild nettle for the determination of simple organic acids, nitrates and phosphates by capillary isotachophoresis, and flavonols, phenolic acids, flavan-3-ols, vitamin B_3_ and trigonelline by high-performance liquid chromatography coupled with mass spectrometry (LC–MS/MS). In addition, the optimization of the extraction process was performed using the Response Surface Methodology. The Principal Component Analysis (PCA) between the nettle samples and organic acids, phenolic acids, flavonols, vitamin B and trigonelline was finally performed. The results should demonstrate whether the use of nettle roots to prepare infusions may be valuable to consumers. In addition, the findings will show whether there is potential for the commercial use of the roots from agricultural nettle cultivation.

## 2. Results and Discussion

### 2.1. Optimization of the Extraction Process

The extraction-optimization process from fresh nettle was performed based on the results obtained from the DPPH and Folin–Ciocalteu methods. A design summary of the Central Composite Design models is presented in Table 1. The optimization was aimed at obtaining the highest levels of antioxidant activity and phenolic content. Information about the ANOVA for the Response Surface Models can be found in the Supplementary Material (Tables S1 and S2 and Figures S1 and S2).

The optimal extraction time was 10 min at 95 °C (Table 2). The results under these extraction conditions in the DPPH and Folin–Ciocalteu methods were 2.5 ± 0.1 mg Trolox 100 mL^−1^ and 3.1 ± 0.2 mg GAE 100 mL^−1^, respectively (*p* > 0.05).

An infusion prepared from nettle leaves (extraction of 1 g of fresh leaves with 110 mL of water in 10 min) was previously found to show 13.5% higher results with the Folin–Ciocalteu method compared to boiling for 5 min, while in the ABTS method, similar antioxidant activity was observed [22]. Furthermore, Elez Garofulic et al. [23] showed that the microwave-assisted extraction (MAE) of nettle leaves can be a very efficient technique for the isolation of individual phenolic compounds. On the other hand, extracts obtained by pressurized liquid extraction (PLE) possessed a higher level of total phenolic compounds as measured by the use of the Folin–Ciocalteu reagent and a higher antioxidant capacity in the ORAC assay than MAE and conventional heat-reflux extraction (H-RE). Nevertheless, although MAE and PLE techniques were more effective compared to H-RE [23] and useful in nettle studies, they are not used for the preparation of infusions intended for consumption.

### 2.2. Capillary Isotachophoresis of Nitrate (III), Phosphate (V) and Organic Acids in Fresh Nettle

Among the short-chain organic acids, citric and malic acids were found to be predominant in the nettle infusions (Table 3). The aerial parts of the nettle had a higher level of malic acid than the roots. Minor amounts of phosphate and oxalic acid were determined in the samples, but their content did not depend on the part of the plant they originated from (Table 3). Formic and tartaric acids were not detected in the nettle infusions, nor nitrate (III) and aspartic acid, except for the root sample IIa, where the last two were detected below the limit of quantification (data not shown).

Previously, an analysis of Polish dried nettle showed that citric acid was the dominant compound (5.72 mg g^−1^), and malic acid was determined in a much lower amount (0.0003 mg g^−1^), but information about the aerial parts or only the leaves was not provided [24]. Other organic acids such as maleic, gluconic and fumaric were also determined.

Interestingly, formic acid, which is widely known as a pain-inducing compound in nettle, was not found in the present study. Nevertheless, Fu et al. [25] estimated that its concentration in the liquid taken from the stinging hair was only 9.4 µg mL^−1^. The main compounds responsible for pain were oxalic acid and tartaric acid found at 1.2 mg mL^−1^ and 14 mg mL^−1^, respectively [25]. A similar concentration of oxalic acid was found in the nettle in the present study (Table 3).

The content of short-chain organic acids, such as citric and formic acid, can influence the sensory aspect of the nettle. Recently, it was found that brewed infusions from fresh or dried nettle leaves showed similar intensities of aroma and flavor. The aroma and flavor of nettle leaf infusions are more burnt and fishy and their taste is more bitter than that prepared from spinach leaves [26]. Furthermore, the level of oxalic acid should be low in the context of nephrolithiasis and Ca deficiency.

### 2.3. LC–MS/MS Determination of Organic Acids, Phenolic Compounds, Vitamin B_3_ and Trigonelline in Fresh Nettle

Of all the acids determined by the LC–MS/MS technique in the present study, the highest amounts were found for 3-caffeoylquinic acid, succinic, and quinic acids (Table 4). The results of 3-caffeoylquinic and quinic acids were comparable to those previously reported [9,10,23]. On the contrary, gallic, caffeic, and sinapic acids were not detected. Repajić et al. [9] reported that the level of gallic acid was between 2.4 and 299 µg g^−1^ in dried leaves and from not detected to 91.2 µg g^−1^ in stems; caffeic acid content was from 209 to 2144 µg g^−1^ in leaves and 53 to 82 µg g^−1^ in stems; sinapic acid from 2.9 to 83.7 µg g^−1^ in leaves and from 1.5 to 79.7 µg g^−1^ in stems.

The highest amounts of succinic, quinic, protocatechuic, and 3-caffeoylquinic acids were found in the leaves and the lowest (up to a few hundred times lower) in the root (Table 4). No such dependence was observed for *p*-coumaric acid. However, a considerable variation was found between the samples of different origins. A similar differentiation in *p*-coumaric acid content depending on the growing conditions was reported by Pinneli et al. [12].

Otles and Yalcin [27] found similar amounts of syringic acid in the roots (0.13–4.31 mg g^−1^ fresh nettle roots), stems 1.20–2.63 (mg g^−1^ fresh nettle stems) and a very wide range of results for leaves (0–341 mg g^−1^ fresh nettle leaves) in 19 nettles from 4 regions of Turkey.

The highest contents of the flavonols analyzed, especially rutin, were determined in the aerial parts of the plant (Table 5). These results are comparable to previous analyses [9,10]. On the contrary, flavan-3-ols, determined as (+)-catechin, (−)-catechin 3-gallate, (−)-epicatechin, (−)-epicatechin-3-gallate, (−)-epigallocatechin 3-gallate, (−)-gallocatechin, and (−)-gallocatechin 3-gallate were not detected. Repajić et al. [9] reported that the total amount of (−)-epigallocatechin 3-gallate, (−)-epicatechin-3-gallate, (+)-catechin, and (−)-epicatechin were between 9.0 and 103 μg g^−1^ of dried mass for leaves of different origin in Croatia and at different stages of harvest.

The aerial parts of samples IV, VI, VIII, IX, and XIV, which contained considerably more rutin and 3-caffeoylquinic acid than other samples, were harvested in sunny places (Table 4 and Table 5). Otles and Yalcin [27] reported that rutin and other phenolic compounds were the dominant compounds in nettle leaves, and the highest level of these compounds was mainly determined in the samples from the Mediterranean region of Turkey.

The differences between the phenolic compounds of Polish and other European nettles such as those from Serbia [6] and Croatia [9] depend not only on the drying process, but also on the soil, after additional harvesting processes and the extraction process.

The presence of vitamin B_3_ determined as nicotinamide and nicotinic acid was found in all parts of the plant, but mainly in the leaves (Table 5).

The highest level of trigonelline for sample IV was 108.3 µg g^−1^ but the difference between the aerial parts of the nettle was huge (9.4–108.3 µg g^−1^). To the best of our knowledge, this is the first time that this alkaloid has been separately determined in the root, stem, and leaves after water extraction of the nettle parts. Previously, Grauso et al., (2019) using ^1^H-NMR analysis, found that the methanol–water extract of nettle leaves contained trigonelline [28]. However, the present study showed that differentiation between the root, stem, and leaves is important, as (except for one sample) nettles contain considerably more trigonelline in the leaves than in the root.

### 2.4. Principal Component Analysis

The Principal Component Analysis of the root, stem, leaves and aerial parts of fresh nettle was carried out taking into account the content of organic acids, flavonols, phenolic acids, trigonelline, nicotinamide, and nicotinic acid.

**Figure 1:**
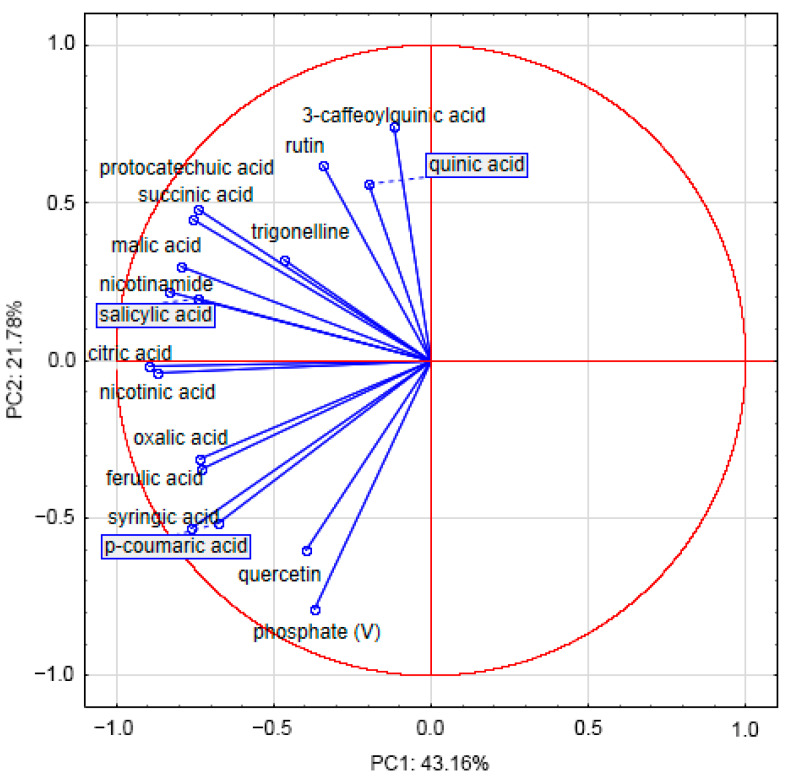
Projection—variable representation onto a plane.

The projection (Figure 1) shows that the PC1 (axis 1) is negatively correlated with almost all the variables. The observations with the largest negative coordinate on the horizontal axis correspond to the most important compounds. Along the vertical axis, quercetin and phosphate(V) are in opposition to 3-caffeoylquinic and quinic acids.

**Figure 2:**
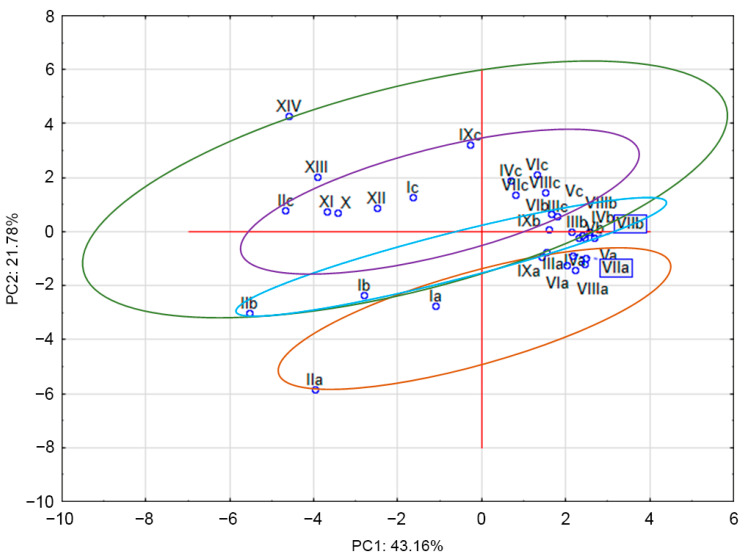
PCA of fresh nettle leaves (samples Ic–IXc), stems (Ib–IXb), aerial parts (leaves with stems X–XIV), and roots (samples Ia–IXa).

The PCA shows the differences between the samples (Figure 2). The sum of PC1 and PC2 was 64.94%. Two separate clusters were found; the one obtained for the aerial parts, leaves, and stems was separated from that found for roots. However, clusters due to stems and leaves could not be completely separated due to the large variation in leaf extracts. To show broader differences between the roots, stems, and leaves, additional analyses may be performed, the results of which can be applied in practice.

## 3. Materials and Methods

### 3.1. Material and Optimization Method

**Figure 3:**
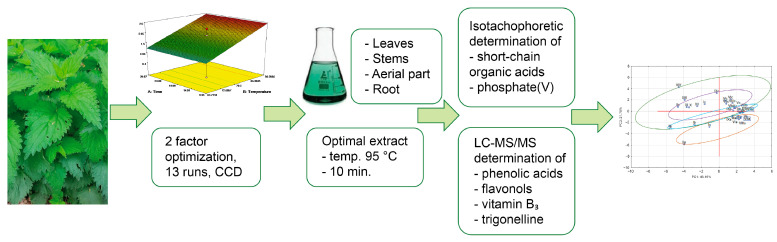
Workflow diagram.

The roots, stems, and leaves of freshly harvested nettles (*Urtica dioica* L.) from the vicinity of Poznań (I–XIV) were used as the material for the study (Table 6). To select the conditions for the extraction of phenolic compounds and the antioxidant activity, 13 nettle samples were extracted with deionized water using the Response Surface Methodology [29]. For this purpose, spectrophotometric tests were performed with DPPH (2,2′-diphenyl-1-picrylhydrazyl) and Folin–Ciocalteu reagents [30]. The workflow diagram can be found in Figure 3.

### 3.2. Isotachophoretic Method

An Electrophoretic Analyzer EA 100 (Villa Labeco, Slovak Republic) was used for the isotachophoretic separations. It was equipped with a column coupling system consisting of two capillaries made of fluorinated ethylene–propylene copolymer: the preseparation capillary (160 mm × 0.8 mm I.D.) and the analytical capillary (160 mm × 0.3 mm I.D.). The first one was connected to the analytical capillary via the bifurcation block. The analyzer was equipped with a sample valve of 30 µL fixed volume. Conductivity detectors were located on both columns 40 mm from the outlet ends. The separations were carried out at ambient temperature. The isotachopherograms were evaluated using a personal computer software package provided with the analyzer. The leading electrolyte was 10 mmol L^−1^ hydrochloric acid containing 1% Triton X-100 and adjusted with β-alanine to pH 3.0. The terminating electrolyte was 5 mmol L^−1^ propionic acid (pH 3.5). The driving current in the preseparation capillary was 250 µA. The initial driving current in the analytical capillary was 50 µA [30]. The structures of the compounds determined using isotachophoresis are presented in the Supplementary Material—Table S3. The method quality control parameters are included in the Supplementary material—Table S4.

### 3.3. LC–MS/MS

The LC–MS/MS analysis of the infusions was performed on the UltiMate 3000 RSLC chromatographic system from Dionex (Sunnyvale, CA, USA) coupled with the API 4000 QTRAP triple quadrupole mass spectrometer from AB Sciex (Foster City, CA, USA) equipped with the Turbo Ion Spray source. The system was equipped with a Kinetex Evo C18 column (150 mm × 2.1 mm I.D.; 2.6 µm) from Phenomenex (Torrance, CA, USA) that was thermostated at 35 °C. The injection volume was 5 µL. The analysis was carried out in a gradient mode with 0.1% formic acid in water serving as phase A and acetonitrile as phase B (Supplementary Material—Table S5). A constant flow rate of 0.3 mL min^−1^ was used throughout the run. The eluate from the column was directed to the source that operated in a negative or positive mode (depending on the analyte). The dwell time for each transition was set to 50 ms and the transitions for the analytes are given in the Supplementary Material (Table S5). The ionization polarities, mass transitions, and collision energies of particular analytes are summarized in the Supplementary Material (Table S6). The structures of the compounds determined using LC-MS/MS are presented in the Supplementary Material—Table S3. The method quality control parameters are included in the Supplementary Material—Table S7.

### 3.4. Statistical Analysis

The results were expressed as mean ± standard deviation (at least three replicates). The optimization experiments were planned according to the central composite design (CCD) with a factorial design that contained four factorial points, four axial points, and five central points. The adequacy of the models was determined by evaluating the lack of fit, the coefficient of determination R^2^, and adjusted R^2^, and the Fisher test value (F value) was obtained from the analysis of variance (ANOVA). The Principal Component Analysis (PCA) between nettle samples and organic acids, phenolic acids, flavonols, vitamin B, and trigonelline was performed. The experimental data were analyzed using the Statistica 13.0 program.

## 4. Conclusions

In summary, the highest amounts of phenolic compounds (especially 3-caffeoylquinic acid and rutin) were determined in samples IV, VI, VIII, IX and XIV, which may be related to increased sunlight, as the other samples were taken from shaded areas. The lowest content of polyphenols (3-caffeoylquinic acid or rutin), as well as succinic and quinic acids, and trigonelline was determined in the root of the plant. As a result, the Principal Component Analysis also demonstrated differences in the content of bioactive components between the roots and the aerial parts of the nettle.

## Figures and Tables

**Table 1:** Design summary of models obtained for extraction from nettle.

Model											
**Factor**	**Name**	**Units**	**Type**	**Low** **Actual**	**High Actual**	**Low Coded**	**High Coded**	**Mean**	**Standard Deviation**		
A	Time	min	Numeric	9.93	29.87	−1.000	1.000	19.99	7.83		
B	Temperature	°C	Numeric	65.71	93.29	−1.000	1.000	79.92	10.90		
**Response**	**Name**	**Units**	**Observations**	**Analysis**	**Min.**	**Max.**	**Mean**	**Std. Dev.**	**Ratio**	**Trans**	**Model**
Y1	DPPH	mg Trolox 100 mL^−1^	13	Polynomial	0.50	2.55	1.90	0.62	5.14	None	Linear
Y2	Folin– Ciocalteu	mg GAE 100 mL^−1^	13	Polynomial	1.01	2.48	1.74	0.44	2.44	None	Linear

**Table 2:** The optimization parameters and responses.

No.	Run	Parameters		Results	
		Time (min)	Temp (°C)	DPPH (mg Trolox 100 mL^−1^)	Folin–Ciocalteu (mg GAE 100 mL^−1^)
1	11	10	66	0.5 ± 0.1	1.3 ± 0.1
2	5	30	66	1.0 ± 0.1	1.3 ± 0.1
3	9	10	95	2.5 ± 0.2	2.9 ± 0.2
4	2	30	95	2.3 ± 0.1	2.4 ± 0.1
5	3	6	80	2.2 ± 0.1	1.8 ± 0.2
6	10	34	80	2.2 ± 0.1	1.5 ± 0.1
7	12	20	60	1.0 ± 0.1	1.0 ± 0.1
8	13	20	99	2.5 ± 0.2	2.5 ± 0.2
9	6	20	80	1.7 ± 0.1	1.7 ± 0.1
10	4	20	80	1.9 ± 0.2	1.7 ± 0.1
11	7	20	80	2.2 ± 0.2	1.7 ± 0.1
12	1	20	80	2.2 ± 0.2	1.7 ± 0.2
13	8	20	80	2.2 ± 0.2	1.8 ± 0.1

GAE—gallic acid equivalent

**Table 3:** Phosphate (V) and organic acid contents of nettle determined with capillary isotachophoresis (mg g^−1^ of fresh weight); samples I–IX: a—root; b—stem; c—leaves; X–XIV—aerial parts of nettle.

Nettle Sample		Phosphate (V)	Oxalic Acid	Citric Acid	Malic Acid
I	a (root)	5.70 ± 0.22	0.99 ± 0.02	6.45 ± 0.32	5.04 ± 0.22
	b (stem)	5.94 ± 0.17	0.58 ± 0.01	13.08 ± 1.02	7.36 ± 0.33
	c (leaves)	0.72 ± 0.04	1.05 ± 0.03	7.40 ± 0.41	7.28 ± 0.24
II	a (root)	10.31 ± 0.12	1.83 ± 0.03	9.54 ± 0.21	2.37 ± 0.12
	b (stem)	9.43 ± 0.31	0.65 ± 0.01	7.49 ± 0.19	3.51 ± 0.11
	c (leaves)	1.97 ± 0.05	0.90 ± 0.02	12.17 ± 0.71	9.13 ± 0.39
III	a (root)	0.92 ± 0.03	0.52 ± 0.12	2.77 ± 0.12	1.69 ± 0.19
	b (stem)	0.87 ± 0.02	0.31 ± 0.02	2.04 ± 0.03	1.38 ± 0.23
	c (leaves)	0.40 ± 0.01	0.31 ± 0.03	1.45 ± 0.21	1.91 ± 0.20
IV	a (root)	4.61 ± 0.07	0.55 ± 0.02	0.26 ± 0.03	0.47 ± 0.02
	b (stem)	0.13 ± 0.02	0.22 ± 0.01	3.10 ± 0.05	1.53 ± 0.05
	c (leaves)	0.53 ± 0.02	0.44 ± 0.04	4.14 ± 0.06	0.86 ± 0.03
V	a (root)	2.20 ± 0.04	0.48 ± 0.05	0.69 ± 0.03	0.44 ± 0.02
	b (stem)	1.02 ± 0.03	0.31 ± 0.03	1.26 ± 0.04	1.55 ± 0.04
	c (leaves)	1.00 ± 0.02	0.56 ± 0.04	1.64 ± 0.05	1.07 ± 0.03
VI	a (root)	2.70 ± 0.05	0.56 ± 0.03	0.76 ± 0.03	1.24 ± 0.02
	b (stem)	1.41 ± 0.03	0.37 ± 0.05	3.14 ± 0.04	2.10 ± 0.06
	c (leaves)	1.23 ± 0.02	0.48 ± 0.04	2.72 ± 0.05	1.75 ± 0.04
VII	a (root)	3.58 ± 0.06	0.44 ± 0.03	1.23 ± 0.03	0.84 ± 0.03
	b (stem)	1.50 ± 0.02	0.25 ± 0.02	1.58 ± 0.04	1.50 ± 0.03
	c (leaves)	1.59 ± 0.03	0.49 ± 0.03	2.35 ± 0.05	1.64 ± 0.03
VIII	a (root)	3.00 ± 0.04	0.34 ± 0.02	0.85 ± 0.03	0.37 ± 0.01
	b (stem)	2.04 ± 0.03	0.26 ± 0.02	3,39 ± 0.06	1.87 ± 0.03
	c (leaves)	3.12 ± 0.02	0.32 ± 0.02	3.63 ± 0.06	2.41 ± 0.04
IX	a (root)	1.68 ± 0.02	0.54 ± 0.04	5.23 ± 0.05	3.52 ± 0.04
	b (stem)	2.35 ± 0.02	0.24 ± 0.01	4.72 ± 0.05	4.06 ± 0.06
	c (leaves)	1.68 ± 0.01	0.54 ± 0.03	5.23 ± 0.04	3.52 ± 0.05
X		0.74 ± 0.01	0.96 ± 0.02	11.60 ± 0.12	12.84 ± 0.75
XI		1.90 ± 0.04	0.88 ± 0.01	10.31 ± 0.15	11.88 ± 0.71
XII		1.41 ± 0.03	0.66 ± 0.01	8.01 ± 0.10	6.01 ± 0.54
XIII		0.75 ± 0.02	0.99 ± 0.02	9.54 ± 0.09	12.99 ± 0.81
XIV		0.59 ± 0.01	0.74 ± 0.01	7.68 ± 0.09	9.55 ± 0.54

**Table 4:** Organic and phenolic acids in nettle determined with the use of LC–MS/MS (µg g^−1^ of fresh weight); samples I–IX: a—root; b—stem; c—leaves; X–XIV—aerial parts of nettle.

Nettle Sample		Succinic Acid	Salicylic Acid	Syringic Acid	Quinic Acid	Protocatechuic Acid	3-Caffeoylquinic Acid	*p*-Coumaric Acid	Ferulic Acid
I	a	20.0 ± 0.8	not detected	0.17 ± 0.02	20.5 ± 0.7	0.13 ± 0.01	2.13 ± 0.02	4.85 ± 0.23	1.44 ± 0.03
	b	96.6 ± 1.1	not detected	0.39 ± 0.03	135.5 ± 1.7	2.08 ± 0.12	75.7 ± 2.5	10.35 ± 0.82	0.95 ± 0.02
	c	359.0 ± 5.4	not detected	0.14 ± 0.01	242.6 ± 2.1	4.27 ± 0.10	308.8 ± 4.1	0.25 ± 0.01	not detected
II	a	13.5 ± 0.4	0.27 ± 0.01	0.73 ± 0.04	59.9 ± 1.0	0.52 ± 0.02	11.7 ± 1.1	6.39 ± 0.31	1.20 ± 0.05
	b	473.1 ± 5.1	3.72 ± 0.02	1.02 ± 0.02	86.4 ± 1.5	3.39 ± 0.13	113.6 ± 3.2	8.30 ± 0.34	1.88 ± 0.06
	c	237.5 ± 2.2	2.17 ± 0.02	0.37 ± 0.02	186.5 ± 2.1	7.11 ± 0.21	200.1 ± 3.5	1.21 ± 0.02	0.53 ± 0.01
III	a	19.7 ± 2.0	1.99 ± 0.01	0.14 ± 0.01	5.0 ± 0.4	0.13 ± 0.01	9.0 ± 0.6	0.90 ± 0.03	0.34 ± 0.03
	b	30.3 ± 3.2	0.24 ± 0.02	0.01 ± 0.01	10.1 ± 1.5	0.18 ± 0.01	202.0 ± 2.7	0.66 ± 0.01	0.16 ± 0.03
	c	52.7 ± 4.2	0.16 ± 0.01	0.02 ± 0.01	89.2 ± 3.2	0.74 ± 0.02	877.5 ± 4.9	0.30 ± 0.01	0.37 ± 0.03
IV	a	11.3 ± 1.1	0.01 ± 0.01	0.07 ± 0.01	7.0 ± 0.8	0.18 ± 0.01	2.0 ± 0.3	0.38 ± 0.01	0.41 ± 0.03
	b	43.4 ± 3.5	0.05 ± 0.01	0.01 ± 0.01	42.2 ± 2.5	0.05 ± 0.01	121.63 ± 3.6	0.28 ± 0.01	0.09 ± 0.03
	c	206.1 ± 4.2	0.13 ± 0.01	0.01 ± 0.01	313. 3 ± 4.3	0.34 ± 0.01	1212.24 ± 8.5	0.32 ± 0.01	0.42 ± 0.03
V	a	8.0 ± 0.5	0.01 ± 0.01	0.05 ± 0.01	1.6 ± 0.3	0.09 ± 0.01	1.98 ± 0.5	0.29 ± 0.01	0.38 ± 0.03
	b	38.2 ± 3.3	0.11 ± 0.01	0.01 ± 0.01	28.0 ± 2.1	0.07 ± 0.01	135.5 ± 1.9	0.20 ± 0.01	0.12 ± 0.01
	c	71.9 ± 4.1	0.09 ± 0.01	0.01 ± 0.01	156.2 ± 3.4	0.66 ± 0.01	824.5 ± 6.4	0.28 ± 0.01	0.28 ± 0.01
VI	a	16.9 ± 1.6	0.01 ± 0.01	0.12 ± 0.01	6.3 ± 0.5	0.13 ± 0.01	1.8 ± 0.4	0.50 ± 0.01	0.62 ± 0.01
	b	63.3 ± 3.9	0.21 ± 0.01	0.01 ± 0.01	33.0 ± 1.3	0.62 ± 0.01	292.6 ± 3.7	0.36 ± 0.01	0.14 ± 0.01
	c	112.4 ± 5.1	0.23 ± 0.01	0.01 ± 0.01	377.8 ± 3.6	0.53 ± 0.01	1991.8 ± 9.1	0.42 ± 0.01	0.25 ± 0.01
VII	a	10.8 ± 1.8	0.03 ± 0.01	0.03 ± 0.01	8.7 ± 0.7	0.15 ± 0.01	1.7 ± 0.4	0.35 ± 0.01	0.34 ± 0.01
	b	28.6 ± 1.9	0.08 ± 0.01	0.01 ± 0.01	18.4 ± 1.4	0.24 ± 0.01	97.5 ± 2.3	0.32 ± 0.01	0.20 ± 0.01
	c	83.8 ± 3.1	0.36 ± 0.01	0.01 ± 0.01	340.0 ± 4.1	0.55 ± 0.01	751.0 ± 4.8	0.31 ± 0.01	0.63 ± 0.01
VIII	a	5.4 ± 0.4	0.01 ± 0.01	0.08 ± 0.01	4.2 ± 0.4	0.11 ± 0.01	1.0 ± 0.1	0.54 ± 0.01	0.52 ± 0.01
	b	52.0 ± 2.4	0.23 ± 0.01	0.01 ± 0.01	46.3 ± 2.6	0.20 ± 0.01	198.4 ± 2.9	0.45 ± 0.01	0.16 ± 0.01
	c	97.5 ± 3.7	0.01 ± 0.01	0.01 ± 0.01	558.3 ± 4.1	0.50 ± 0.01	1338.8 ± 6.3	0.25 ± 0.01	0.22 ± 0.01
IX	a	19.0 ± 2.0	0.26 ± 0.01	0.10 ± 0.01	11.1 ± 1.0	0.14 ± 0.01	2.3 ± 0.4	0.73 ± 0.01	0.69 ± 0.02
	b	86.9 ± 3.3	0.40 ± 0.01	0.01 ± 0.01	52.7 ± 2.9	0.22 ± 0.01	174.7 ± 3.7	0.62 ± 0.01	0.22 ± 0.01
	c	315.4 ± 4.1	0.41 ± 0.01	0.01 ± 0.01	690.1 ± 4.8	1.94 ± 0.02	2126.5 ± 7.9	0.68 ± 0.01	0.44 ± 0.01
X		247.5 ± 2.1	1.49 ± 0.01	0.28 ± 0.01	193.2 ± 2.3	4.05 ± 0.11	567.1 ± 5.1	4.12 ± 0.21	0.74 ± 0.03
XI		253.1 ± 2.7	1.50 ± 0.02	0.22 ± 0.01	305.9 ± 3.2	4.51 ± 0.13	481.2 ± 4.8	3.76 ± 0.20	1.02 ± 0.04
XII		189.8 ± 2.3	1.92 ± 0.03	0.19 ± 0.01	109.5 ± 1.5	4.24 ± 0.09	838.6 ± 5.9	1.19 ± 0.07	1.54 ± 0.04
XIII		373.7 ± 3.3	2.60 ± 0.04	0.25 ± 0.02	150.7 ±1.7	6.11 ± 0.13	749.0 ± 5.3	1.22 ± 0.06	0.80 ± 0.02
XIV		348.4 ± 3.5	2.99 ± 0.05	0.14 ± 0.01	94.8 ± 1.2	14.64 ± 1.01	1300 ± 13	2.46 ± 0.12	0.93 ± 0.01

**Table 5:** Flavonols, vitamin B_3_ and trigonelline content in nettle determined with the use of LC–MS/MS (µg g^−1^ of fresh weight); samples I–IX: a—root; b—stem; c—leaves; X–XIV—aerial parts of nettle.

Nettle Sample		Kaempferol	Quercetin	Rutin	Nicotinamide	Nicotinic Acid	Trigonelline
I	a	not detected	0.53 ± 0.02	0.64 ± 0.01	3.77 ± 0.07	4.07 ± 0.08	17.19 ± 0.58
	b	not detected	0.40 ± 0.01	49.38 ± 1.12	4.85 ± 0.08	4.84 ± 0.09	41.41 ± 1.23
	c	not detected	0.25 ± 0.01	12.36 ± 0.71	12.91 ± 0.41	3.57 ± 0.07	40.63 ± 1.19
II	a	not detected	4.71 ± 0.11	6.33 ± 0.32	4.22 ± 0.12	3.18 ± 0.05	79.30 ± 1.34
	b	not detected	1.11 ± 0.03	347.7 ± 4.1	5.49 ± 0.10	5.00 ± 0.09	34.22 ± 1.02
	c	not detected	0.41 ± 0.01	46.7 ± 1.8	14.32 ± 0.55	10.65 ± 1.12	72.27 ± 1.52
III	a	0.34 ± 0.01	0.22 ± 0.01	3.31 ± 0.09	0.52 ± 0.06	0.03 ± 0.01	8.64 ± 0.21
	b	0.22 ± 0.01	0.14 ± 0.02	144.94 ± 5.13	0.41 ± 0.05	not detected	26.44 ± 1.03
	c	1.27 ± 0.03	0.91 ± 0.03	346.63 ± 6.81	1.18 ± 0.22	0.22 ± 0.02	37.58 ± 1.06
IV	a	0.23 ± 0.02	0.06 ± 0.01	0.74 ± 0.12	0.41 ± 0.11	0.47 ± 0.03	13.24 ± 0.09
	b	0.19 ± 0.01	0.12 ± 0.02	33.43 ± 2.11	0.21 ± 0.04	not detected	24.10 ± 0.08
	c	0.28 ± 0.02	0.28 ± 0.01	343.82 ± 5.81	1.57 ± 0.24	0.72 ± 0.22	108.26 ± 2.53
V	a	0.12 ± 0.01	0.04 ± 0.01	0.30 ± 0.03	0.37 ± 0.02	0.20 ± 0.03	14.25 ± 0.79
	b	0.25 ± 0.01	0.05 ± 0.01	86.64 ± 4.32	not detected	not detected	12.93 ± 0.81
	c	0.76 ± 0.03	0.18 ± 0.02	270.35 ± 2.89	1.76 ± 0.12	0.81 ± 0.06	40.11 ± 1.21
VI	a	0.11 ± 0.01	0.0 2± 0.01	0.27 ± 0.2	0.48 ± 0.03	0.22 ± 0.01	13.36 ± 0.83
	b	0.10 ± 0.01	0.03 ± 0.01	148.23 ± 4.1	0.51 ± 0.04	not detected	18.46 ± 1.01
	c	0.21 ± 0.01	0.06 ± 0.01	451.46 ± 1.8	1.41 ± 0.11	0.44 ± 0.02	38.94 ± 1.34
VII	a	0.22 ± 0.02	0.03 ± 0.01	0.26 ± 0.03	0.23 ± 0.01	0.33 ± 0.01	47.90 ± 1.59
	b	0.26 ± 0.02	0.03 ± 0.01	102.81 ± 2.15	0.33 ± 0.01	0.25 ± 0.01	30.76 ± 1.18
	c	0.68 ± 0.03	0.11 ± 0.03	454.49 ± 4.79	1.65 ± 0.12	0.75 ± 0.03	98.91 ± 2.38
VIII	a	0.09 ± 0.01	0.03 ± 0.01	0.97 ± 0.12	0.50 ± 0.01	0.22 ± 0.01	2.85 ± 0.37
	b	0.15 ± 0.02	0.05 ± 0.01	143.82 ± 3.14	0.30 ± 0.01	not detected	9.42 ± 0.88
	c	0.14 ± 0.02	0.03 ± 0.01	205.62 ± 4.24	0.12 ± 0.01	0.65 ± 0.02	29.32 ± 1.12
IX	a	0.12 ± 0.01	0.04 ± 0.01	0.24 ± 0.01	0.67 ± 0.02	0.38 ± 0.01	11.45 ± 0.79
	b	0.18 ± 0.02	0.10 ± 0.02	320.26 ± 3.74	0.21 ± 0.01	0.36 ± 0.01	25.19 ± 1.14
	c	0.49 ± 0.03	0.09 ± 0.01	508.78 ± 4.87	1.47 ± 0.08	1.29 ± 0.02	55.69 ± 2.26
X		not detected	0.25 ± 0.02	157.0 ± 2.9	10.87 ± 0.31	3.75 ± 0.06	36.60 ± 1.01
XI		not detected	0.17 ± 0.01	158.5 ± 3.2	14.22 ± 0.27	4.92 ± 0.07	25.31 ± 0.91
XII		not detected	0.19 ± 0.02	510.4 ± 4.4	11.70 ± 0.61	3.48 ± 0.05	26.60 ± 0.79
XIII		not detected	0.16 ± 0.01	290.2 ± 2.1	8.98 ± 0.32	3.38 ± 0.03	91.41 ± 1.56
XIV		not detected	0.11 ± 0.01	1848 ± 17	8.79 ± 0.21	3.38 ± 0.04	70.31 ± 1.12

**Table 6:** Sample type and sampling location in the vicinity of Poznań, Great Poland region (Poland).

Sample	Sample Type	Origin
Ia	Root	Ditch I
Ib	Stem	Ditch I
Ic	Leaves	Ditch I
IIa	Root	Garden I
IIb	Stem	Garden I
IIc	Leaves	Garden I
IIIa	Root	Ditch II
IIIb	Stem	Ditch II
IIIc	Leaves	Ditch II
IVa	Root	Garden II
IVb	Stem	Garden II
IVc	Leaves	Garden II
Va	Root	Field I
Vb	Stem	Field I
Vc	Leaves	Field I
VIa	Root	Field II
VIb	Stem	Field II
VIc	Leaves	Field II
VIIa	Root	Field III
VIIb	Stem	Field III
VIIc	Leaves	Field III
VIIIa	Root	Field IV
VIIIb	Stem	Field IV
VIIIc	Leaves	Field IV
IXa	Root	Forest I
IXb	Stem	Forest I
IXc	Leaves	Forest I
X	Aerial part	Forest II
XI	Aerial part	Field V
XII	Aerial part	Ditch III
XIII	Aerial part	Forest III
XIV	Aerial part	Ditch IV

## Data Availability

Not applicable.

## Supplementary Materials

The following supporting information can be downloaded at: https://www.mdpi.com/article/10.3390/molecules27134242/s1, Figure S1. Model graph for fresh nettle obtained for the DPPH method; Figure S2. Model graph for fresh nettle obtained for the Folin-Ciocalteu method; Supplementary Table S1. ANOVA for Response Surface Linear Model for fresh nettle leaves for the DPPH method; Supplementary Table S2. ANOVA for Response Surface Linear Model for fresh nettle leaves for the Folin-Ciocalteu method; Supplementary Table S3. Linearity, limit of detection and limit of quantitation in the ITP method; Supplementary Table S4. LC mobile phase gradient and MS source parameters; Supplementary Table S5. Retention times and mass spectrometer parameters applied for the determination of compounds; Supplementary Table S6. Linearity, limit of detection and limit of quantitation in the LC-MS/MS method.
