# Supplementary material for: Variation in the Content of Bioactive Compounds in Infusions Prepared from Different Parts of Wild Polish Stinging Nettle (Urtica dioica L.)
Source: Molecules. 2022 Jun 30;27(13):4242. doi: 10.3390/molecules27134242 (PMC9268169; doi:10.3390/molecules27134242)
Supplement: Supplementary file 1 [file molecules-27-04242-s001.zip › molecules-1782979-supplementary.pdf]

*Article*

# Variation in the Content of Bioactive Compounds in Infusions Prepared from Different Parts of Wild Polish Stinging Nettle (*Urtica Dioica* L.)

**Magdalena Jeszka-Skowron [1,*], Agnieszka Zgoła-Grześkowiak [1], Robert Frankowski [1], Tomasz Grześkowiak [1] and Anna Maria Jeszka [2]**

[1] Institute of Chemistry and Technical Electrochemistry, Poznan University of Technology, Berdychowo 4, 60-965 Poznań, Poland; agnieszka.zgola-grzeskowiak@put.poznan.pl (A.Z.-G.); robert.frankowski@put.poznan.pl (R.F.); civ@tlen.pl (T.G.)

[2] Institute of Socio-Economics, Poznan University of Economics and Business, Al. Niepodległości 10, 61-875 Poznań, Poland; anna.jeszka@ue.poznan.pl

* Correspondence: magdalena.jeszka-skowron@put.poznan.pl; Tel.: +48-665-3347

**Table S1.** ANOVA for Response Surface Linear Model for fresh nettle leaves for the DPPH method.

| **Source** | **Sum of Squares** | **df** | **Mean Square** | **F Value** | ***p*-Value Prob > F** | |
|---|---|---|---|---|---|---|
| Model | 3.67 | 1 | 3.67 | 30.44 | 0.0002 | significant |
| B-Temperature | 3.67 | 1 | 3.67 | 30.44 | 0.0002 | |
| Residual | 1.33 | 11 | 0.12 | | | |
| Lack of Fit | 1.04 | 7 | 0.15 | 2.04 | 0.2557 | not significant |
| Pure Error | 0.29 | 4 | 0.072 | | | |
| Cor Total | 4.99 | 12 | | | | |

| | | | |
|---|---|---|---|
| Std. Dev. | 0.35 | R-Squared | 0.7346 |
| Mean | 1.90 | Adj R-Squared | 0.7104 |
| C.V. % | 18.30 | Pred R-Squared | 0.6210 |
| PRESS | 1.89 | Adeq Precision | 13.957 |

| Factor | Coefficient Estimate | df | Standard Error | 95% CI Low | 95% CI High | VIF |
|---|---|---|---|---|---|---|
| Intercept | 1.88 | 1 | 0.096 | 1.66 | 2.09 | |
| B-Temperature | 0.67 | 1 | 0.12 | 0.40 | 0.94 | 1.00 |

**Table S2.** ANOVA for Response Surface Linear Model for fresh nettle leaves for the Folin-Ciocalteu method.

| **Source** | **Sum of Squares** | **df** | **Mean Square** | **F Value** | ***p*-Value Prob > F** | |
|---|---|---|---|---|---|---|
| Model | 2.33 | 1 | 2.33 | 180.59 | < 0.0001 | significant |
| B-Temperature | 2.33 | 1 | 2.33 | 180.59 | < 0.0001 | |
| Residual | 0.14 | 11 | 0.013 | | | |
| Lack of Fit | 0.13 | 7 | 0.019 | 5.91 | 0.0527 | not significant |
| Pure Error | 0.013 | 4 | 3.135E-003 | | | |
| Cor Total | 2.48 | 12 | | | | |

| | | | | | | |
|---|---|---|---|---|---|---|
| Std. Dev. | 0.11 | R-Squared | 0.9426 | | | |
| Mean | 1.74 | Adj R-Squared | 0.9374 | | | |
| C.V. % | 6.52 | Pred R-Squared | 0.9252 | | | |
| PRESS | 0.19 | Adeq Precision | 33.995 | | | |

| Factor | Coefficient Estimate | df | Standard Error | 95% CI Low | 95% CI High | VIF |
|---|---|---|---|---|---|---|
| Intercept | 1.73 | 1 | 0.032 | 1.66 | 1.80 | |
| B-Temperature | 0.54 | 1 | 0.040 | 0.45 | 0.62 | 1.00 |

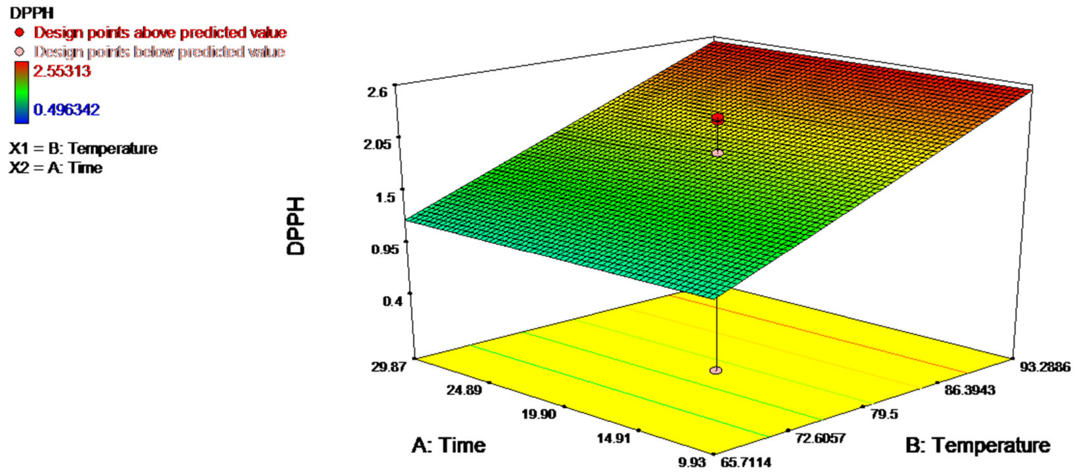


**Figure S1.** Model graph for fresh nettle obtained for the DPPH method.

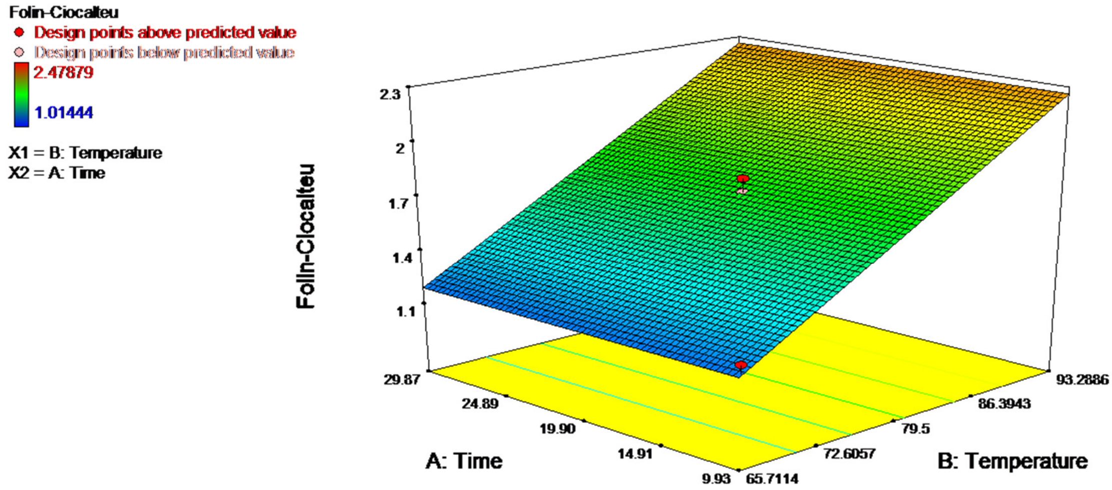


**Figure S2.** Model graph for fresh nettle obtained for the Folin-Ciocalteu method.

**Table S3.** Structures of compounds determined using capillary isotachophoresis (oxalic, citric and succinic acid) and LC-MS/MS (other compounds).

| | | |
|---|---|---|
| Oxalic acid 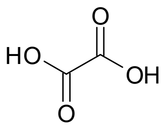 | Citric acid 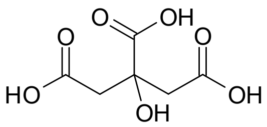 | Succinic acid 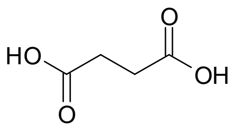 |
| Malic acid 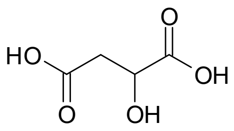 | Salicylic acid 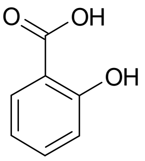 | Syringic acid 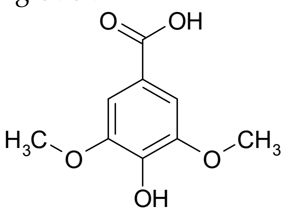 |
| Quinic acid 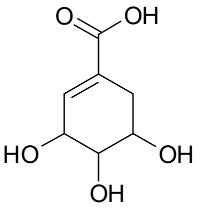 | Protocatechuic acid 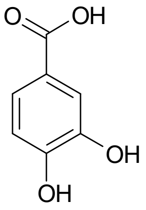 | *p*-coumaric acid 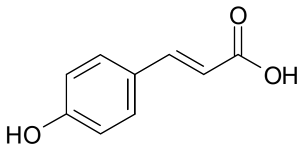 |
| Quercetin 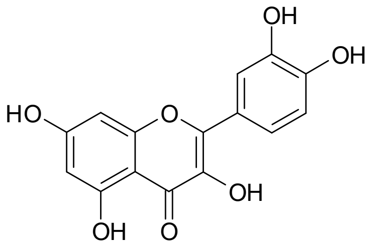 | Ferulic acid 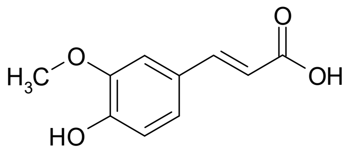 | Kaempferol 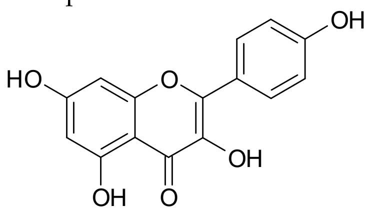 |
| Nicotinamide 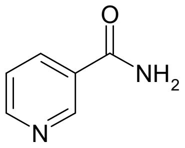 | Nicotinic acid 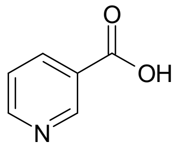 | Trigonelline 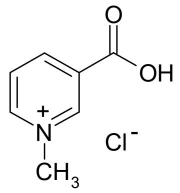 |
| Rutin 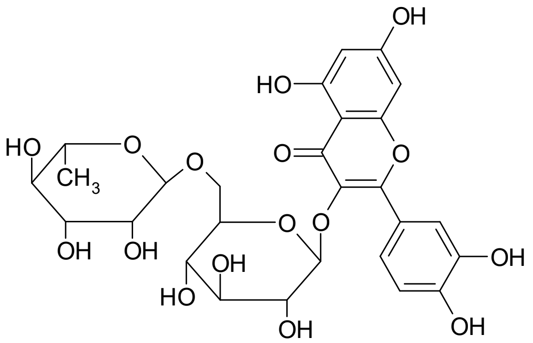 | 3-caffeoylquinic acid 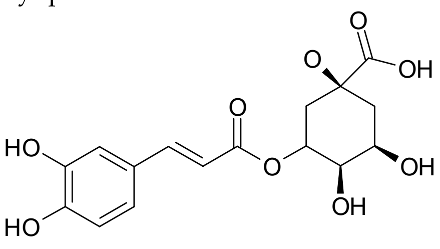 | |

**Table S4.** Linearity, limit of detection and limit of quantitation in the ITP method.

| Analyte | Calibration Curve Range [mg $L^{-1}$] | Correlation Coefficient ($r^2$) | Limit of Detection [mg $L^{-1}$] | Limit of Quantitation [mg $L^{-1}$] |
|---|---|---|---|---|
| Phosphate (V) | 8–500 | 0.9994 | 3 | 8 |
| Oxalic acid | 8–500 | 0.9964 | 3 | 8 |
| Citric acid | 20–500 | 0.9999 | 7 | 20 |
| Malic acid | 20–500 | 0.9996 | 7 | 20 |

**Table S5.** LC mobile phase gradient and MS source parameters.

| Mobile phase Gradient (Percentage of Acetonitrile) | Parameters of Source |
|---|---|
| Negative ionization | |
| 0 min 5%,<br>2 min 5%,<br>5 min 15%,<br>10 min 20%<br>11 min 70%<br>15.5 min 90% | curtain gas 20 psi<br>nebulizer gas 45 psi<br>auxiliary gas 50 psi<br>temperature 450 °C<br>collision gas medium<br>ion spray voltage -4500 V<br>entrance potential -10 V |
| Positive ionization | |
| 0 min 10%,<br>2 min 10%,<br>2.5 min 100%,<br>4 min 100% | curtain gas 20 psi<br>nebulizer gas 45 psi<br>auxiliary gas 45 psi<br>temperature 450 °C<br>collision gas medium<br>ion spray voltage 4500 V<br>entrance potential 10 V |

**Table S6.** Retention times and mass spectrometer parameters applied for the determination of compounds.

| Compound | Retention Time [min] | DP[a] [V] | Analytical Transition | CE[b] [V] | CXP[c] [V] | Confirmatory Transition | CE [V] | CXP [V] |
|---|---|---|---|---|---|---|---|---|
| Negative ionization | | | | | | | | |
| Quinic acid | 1.11 | −55 | 191 → 85 | −31 | −5 | 191 → 93 | −31 | −6 |
| Succinic acid | 1.53 | −31 | 117 → 73 | −17 | −4 | 117 → 99 | −16 | −7 |
| Gallic acid | 1.95 | −45 | 169 → 125 | −22 | −7 | 169 → 95 | −56 | −6 |
| (−)-Gallocatechin | 3.34 | −80 | 305 → 125 | −31 | −6 | 305 → 167 | −30 | −2 |
| Protocatechuic acid | 3.46 | −45 | 153 → 109 | −22 | −5 | 153 → 91 | −37 | −3 |
| (+)-Catechin | 5.95 | −80 | 289 → 245 | −23 | −4 | 289 → 203 | −29 | −3 |
| Chlorogenic acid | 6.12 | −40 | 353 → 191 | −30 | −2 | 353 → 85 | −62 | −5 |
| Caffeic acid | 6.47 | −45 | 179 → 135 | −25 | −5 | 179 → 89 | −47 | −6 |
| Syringic acid | 6.79 | −40 | 197 → 182 | −20 | −2 | 197 → 123 | −34 | −9 |
| (−)-Epicatechin | 6.97 | −80 | 289 → 245 | −23 | −4 | 289 → 203 | −29 | −3 |
| (−)-Epigallocatechin 3-gallate | 7.28 | −80 | 457 → 169 | −24 | −2 | 457 → 305 | −28 | −7 |
| (−)-Gallocatechin 3-gallate | 7.80 | −80 | 457 → 169 | −24 | −2 | 457 → 305 | −28 | −7 |
| *p*-Coumaric acid | 8.07 | −35 | 163 → 119 | −26 | −5 | 163 → 93 | −47 | −6 |
| Ferulic acid | 8.96 | −40 | 193 → 134 | −23 | −5 | 193 → 178 | −19 | −6 |
| Sinapic acid | 9.15 | −40 | 223 → 208 | −20 | −3 | 223 → 164 | −22 | −2 |
| (−)-Epicatechin-3-gallate | 9.42 | −80 | 441 → 169 | −29 | −2 | 441 → 289 | −27 | −5 |
| Rutin | 9.48 | −80 | 609 → 300 | −53 | −6 | 609 → 271 | −80 | −4 |
| (−)-Catechin 3-gallate | 9.86 | −80 | 441 → 169 | −29 | −2 | 441 → 289 | −27 | −5 |
| Salicylic acid | 10.92 | −20 | 137 → 93 | −26 | −20 | 137 → 65 | −40 | −4 |
| Quercetin | 12.38 | −80 | 301 → 151 | −31 | −7 | 301 → 179 | −26 | −2 |
| Kaempferol | 12.53 | −80 | 285 → 117 | −60 | −4 | 285 → 93 | −50 | −6 |
| Positive ionization | | | | | | | | |
| Trigonelline | 1.04 | 50 | 138 → 94 | 30 | 4 | 138 → 92 | 30 | 4 |
| Nicotinamide | 1.08 | 15 | 123 → 80 | 28 | 2 | 123 → 96 | 28 | 4 |
| | | | | | | 123 → 78 | 35 | 4 |
| Nicotinic acid | 1.12 | 30 | 124 → 80 | 30 | 3 | 124 → 96 | 29 | 4 |
| | | | | | | 124 → 78 | 32 | 3 |

[a]DP – declustering potential, [b]CE – collision energy, [c]CXP – cell exit potential.

**Table S7.** Linearity, limit of detection and limit of quantitation in the LC-MS/MS method.

| Analyte | Calibration Curve Range [μg $L^{-1}$] | Correlation Coefficient ($r^2$) | Limit of Detection [μg $L^{-1}$] | Limit of Quantitation [μg $L^{-1}$] |
|---|---|---|---|---|
| Quinic acid | 2–4000 | 0.9998 | 0.76 | 2.54 |
| Succinic acid | 2–1000 | 0.9986 | 0.85 | 2.84 |
| Gallic acid | 2–1000 | 0.9992 | 0.40 | 1.32 |
| (-)-Gallocatechin | 20–4000 | 0.9988 | 0.89 | 2.96 |
| Protocatechuic acid | 2–1000 | 0.9984 | 0.12 | 0.40 |
| (+)-Catechin | 2–4000 | 0.9985 | 0.43 | 1.45 |
| 3-chlorogenic acid | 2–1000 | 0.9986 | 0.23 | 0.76 |
| Syringic acid | 2–4000 | 0.9998 | 0.15 | 0.50 |
| (-)-Epicatechin | 2–1000 | 0.9996 | 0.24 | 0.78 |
| (-)-Epigallocatechin 3-gallate | 20–4000 | 0.9995 | 3.44 | 11.47 |
| (-)-Gallocatechin 3-gallate | 10–4000 | 0.9999 | 2.03 | 6.75 |
| Ferulic acid | 2–4000 | 0.9981 | 0.11 | 0.35 |
| Sinapic acid | 2–1000 | 0.9987 | 0.06 | 0.19 |
| (-)-Epicatechin-3-gallate | 2–4000 | 0.9996 | 0.73 | 2.45 |
| (-)-Catechin 3-gallate | 2–4000 | 0.9993 | 0.82 | 2.74 |
| Salicylic acid | 2–4000 | 0.9991 | 0.46 | 1.53 |
| Quercetin | 2–1000 | 0.9975 | 0.14 | 0.48 |
| Caffeic acid | 2–100 | 0.9996 | 0.51 | 1.71 |
| Rutin | 4–2000 | 0.9998 | 0.12 | 0.40 |
| Kaempferol | 4–400 | 0.9995 | 0.50 | 1.67 |
| *p*-coumaric acid | 0.5–250 | 0.9982 | 0.06 | 0.21 |
| Nicotinamide | 0.5–100 | 1.000 | 0.1 | 0.3 |
| Nicotinic acid | 1–1000 | 0.999 | 0.4 | 1.2 |
| Trigonelline | 0.5–50 | 0.998 | 0.04 | 0.1 |
